# Composite monomer conversion and microhardness using short curing times

**DOI:** 10.1590/0103-644020256598

**Published:** 2025-12-08

**Authors:** Marcelo Giannini, Eduardo F. de Castro, Beatriz Ometto Sahadi, Rodrigo de Castro Albuquerque, Frederick A. Rueggeberg

**Affiliations:** 1 University of Campinas, Piracicaba Dental School, Departamento de Dentística Restauradora, Piracicaba, SP, Brazil; 2 Federal University of Minas Gerais, Departament of Restorative Dentistry, Belo Horizonte, MG, Brazil; 3 Department of Restorative Sciences, Dental College of Georgia, Augusta University, Augusta, GA, USA

**Keywords:** light curing time, polymerization, resin composite, curing light, monomer conversion

## Abstract

This study evaluated how short curing times affect monomer conversion and microhardness of a bulk-fill composite. Two light-curing units (LCUs) were used: a laser LCU (Monet, AMD Lasers) and a multi-peak LED LCU (PowerCure, Ivoclar Vivadent). A bulk-fill composite (Tetric PowerFill, Ivoclar) was cured for 1, 2, or 3 seconds with the laser, and for 3 or 20 seconds with the LED. Monomer conversion was measured via real-time FTIR at the bottom of 4-mm composite cylinders. Microhardness was assessed at 100µm, 1mm, 2mm, 3mm, and 390µm depths. Spectral irradiance, radiant emittance, and radiant exposure of each LCU were recorded using a calibrated system. Data were analyzed using ANOVA and Tukey’s test (α = 0.05). Radiant exposures for the laser LCU were 2.5, 5.0, and 7.5 J/cm² for 1, 2, and 3 seconds, respectively. The LED LCU delivered 9.3 J/cm² (3 s) and 22.3 J/cm² (20 s). Monomer conversion at 4 mm increased with more prolonged exposure for both LCUs. The LED LCU at 20 seconds showed significantly higher conversion than at 3 seconds. Microhardness decreased with depth, regardless of curing method or duration. Monomer conversion at 4 mm was higher with increased curing time, correlating with radiant exposure. Only the 20-second LED exposure provided a bottom-to-top microhardness ratio of 0.84, indicating sufficient curing at depth.

## Resumo

Este estudo avaliou como tempos curtos de fotoativação afetam a conversão de monômeros e a microdureza de um compósito do tipo bulk-fill. Foram utilizadas duas unidades fotopolimerizadoras (UFPs): uma UFP a laser (Monet, AMD Lasers) e uma UFP LED de múltiplos picos (PowerCure, Ivoclar Vivadent). Um compósito bulk-fill (Tetric PowerFill, Ivoclar) foi fotoativado por 1, 2 ou 3 segundos com o laser, e por 3 ou 20 segundos com o LED. A conversão de monomérica foi medida em tempo real por FTIR na base de cilindros de compósito com 4 mm de espessura. A microdureza foi avaliada nas profundidades de 100µm, 1mm, 2mm, 3mm, e 390µm. A irradiância espectral, a emitância radiante e a exposição radiante de cada UFP foram registradas por um sistema calibrado. Os dados foram analisados por ANOVA e teste de Tukey (α = 0,05). As exposições radiantes para a UFP a laser foram de 2,5, 5,0 e 7,5 J/cm² para 1, 2 e 3 segundos, respectivamente. A UFP LED forneceu 9,3 J/cm² (3 s) e 22,3 J/cm² (20 s). A conversão de monômero a 4 mm aumentou com o tempo de exposição para ambas as UFPs. A UFP LED em 20 segundos apresentou conversão significativamente maior do que em 3 segundos. A microdureza diminuiu com a profundidade, independentemente do método ou duração da fotoativação. A conversão monomérica a 4 mm foi maior com o aumento do tempo de fotoativação, correlacionando-se com a exposição radiante. Apenas a exposição de 20 segundos com LED proporcionou uma razão de microdureza base/superfície de 0,84, indicando cura suficiente em profundidade.


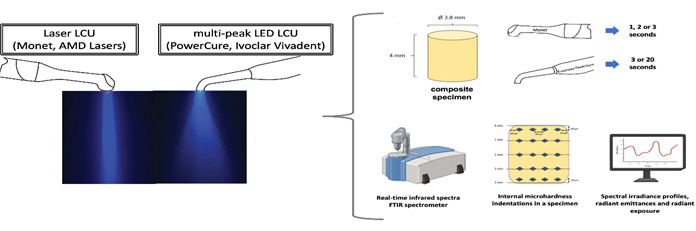


## Introduction

Forty seconds was the recommended light-curing time when the restorative resin composites were cured with visible light generated by halogen lamp curing units. The development of the third-generation of blue LED-curing units that deliver approximately 900 mW/cm^2^ of irradiance yielded the reduction of light curing time to 20 seconds for many commercial composites ^1^. Thus, it has been observed that the reduction of light-curing time is related to the increase in the radiant energy delivered by contemporary LED-curing units ^2^ ^,^ ^3^.

The main drawback of the halogen lamp and LED light curing units (LCUs) has been the light output decreasing with limitations of distance from the composite target, which results in low degree of conversion, particularly when using darker shades, thick increment of traditional resin and bulk-fill composites ^1^ ^,^ ^2^ ^,^ ^3^ ^,^ ^4^ In the 90's, the light-activation of composites with argon-ion laser was proposed in attempt to overcome the low depth of cure and to increase the monomer conversion, but without much success due to costs, possibility of heating and shrinkage stress increasing ^5^ ^,^ ^6^ ^,^ ^7^ ^,^ ^8^.

Current LED-curing units offer different curing modes for clinicians to polymerize the restorative resin composites. The curing mode variations are related to the curing times and their respective irradiances and radiant exposures delivered. In general, the curing modes are called “standard” (irradiance values around 900 mW/cm^2^), “high” (irradiance values ranging from 1,000 to 1,700 mW/cm^2^), and “turbo or extra power” (irradiance values higher than 1,800 mW/cm^2^) ^3^ ^,^ ^9^ ^,^ ^10^ ^,^ ^11^. When using "turbo or extra power" mode, the curing time set by the curing unit manufacturer is very low to avoid the heating effects from the light exposure.

For faster restorative composite curing, dentists can also use the diode laser curing light technology. This laser-curing unit emits a high-power light and coherent collimated beam, with reduced irradiance loss from the emitting tip through direct and indirect restorative materials. The light activation using short curing time and high radiant energy can be desirable for clinicians, as the light curing procedures are faster and easier. Thus, studies have investigated light-curing times and their relationship with the depth of cure of the restorative composite and the effects of high-power lights on the tooth heating and the polymerization shrinkage of the composite ^12^ ^,^ ^13^ ^,^ ^14^ ^,^ ^15^ ^,^ ^16^ ^,^ ^17^ ^,^ ^18^ ^,^ ^19^ ^,^ ^20^ ^,^ ^21^ ^,^ ^22^ ^,^ ^23^ ^,^ ^24^ ^,^ ^25^.

The depth of composite cure has clinical importance due to reports of failures in the proximal cervical margins of deep direct composite restorations (26-28), where the thick resin composite (greater than 4 mm) is placed to restore the tooth. In order to evaluate the effects of high-power lights with short curing times on the monomer conversion at 4 mm depth and the microhardness at different depths of a bulk-fill composite, this study was performed using a diode laser curing light and a high-power LED-curing unit. The research hypotheses were that 1- the composite monomer conversion at 4 mm depth varies according to the LCU types and their curing modes used, and 2- the composite microhardness reduces with increasing depth, regardless of the LCU type and curing mode used.

## Materials and methods

### Curing Light Characterization

A laser diode dental LCU (Monet laser curing light, sn 00315, AMD Lasers, West Jordan, UT, USA) and a multi-peak LED curing light (Bluephase PowerCure, sn 1432002775, Ivoclar Vivadent, Schaan, Liechtenstein) were investigated in this study. The curing tips of each type of light curing unit were kept approximately 3 mm from the integrating sphere/spectroradiometer or the cosine corrector/miniature spectroradiometer apertures during the curing light characterization measurements. The distance of 3 mm to light-cure the resin-based composite was used to simulate the clinical scenario where the tooth cusps limit the proximity of the light-curing unit tip to the composite.

For the laser diode dental light curing unit, the radiant exitance values were too strong when the calibrated 6" integrating sphere (Labsphere Inc., N. Sutton, NH) and connected spectroradiometer (USB 2000+, Ocean Optics, Dunedin, FL) were used. Thus, spectral power readings of the laser diode unit were obtained by placing the curing laser attenuator attachment (a 0.3 ND filter, part of the Monet Aperture Kit, SKU 001-00113, AMD Lasers) over the Monet output lens. Prior to obtaining readings, the exact attenuation of the ND filter was calculated by obtaining spectral irradiance plots of a multi-peak LED curing light in its "High" output mode with and without the filter in place. Five replications of spectral power were obtained in each condition. From this data, it was determined that the attenuator attachment reduced light transmission by 48.9%. This value was later used to back-calculate the emitted laser power if no attenuator was present.

To observe the output variation of the laser diode dental LCU (Monet laser) during the 1-s long exposure, a different spectroradiometer was used, which captured data at a faster rate. For this purpose, high-speed integrated power readings between 430 and 480 nm were captured by measuring light output using a calibrated microradiometer (STS-RAD, Ocean Optics, Dunedin, FL, USA). The output of the laser light was captured during four sequential 1-s long exposures, at a data rate of 43 scans/second using the strip chart mode in the collection software (Spectrasuite, Ocean Optics). Because of differences in light collection areas between the 6” sphere (captured all light emitting from the tip) and the small cosine corrector on the miniature spectroradiometer (0.714 mm diameter), a correction factor of 1.616 was applied to the strip chart power values so that the average power value using the small radiometer would be equivalent to that when using the 6” sphere.

Although the tip diameter of the Monet laser unit is 11 mm, the effective output area of emission is 5.2 mm in diameter^17^. The diameter of the cosine corrector used in this study was 7.1 mm, which captured all of the high-output value of the Monet laser. As long as the spectrometer was not saturated with light, it measured all photons falling on the detector.

Irradiance values using the 6” sphere setup were determined by dividing the measured light power values by the area of light being emitted on a target. For this purpose, two methods of beam measurement were used. The first method used directed the light output onto a small millimeter ruler and noted where the beam started and stopped on the scale, while wearing protective glasses. The second measurement method of the calibrated image of a laser beam analyzer (LBA FireWire with Scorpion, Spiricon Laser Beam Diagnostics, Lohan, UT, USA) when the laser was shown against a 1500-grit ground glass diffuser (item DG2X2-1500, Thorlabs, Newton, NJ, USA). Using the exposure duration measured by the high-speed data acquisition mode, the radiant exposure of light was also determined.

The multi-peak LED LCU (Bluephase PowerCure) presents a 9 mm diameter tip ^18^ ^,^ ^19^ ^,^ ^20^ ^,^ ^21^ and was used in its high output mode for 20 seconds, as well as in its highest output mode for 3 seconds (or “3s cure” mode). The spectral profile of the unit was measured 5 times in each output mode using the calibrated 6” integrating sphere system mentioned above. From these values, the average irradiance, average radiant exposure, and average spectral profile were determined.

### Monomer Conversion Analysis

**Figure 1:**
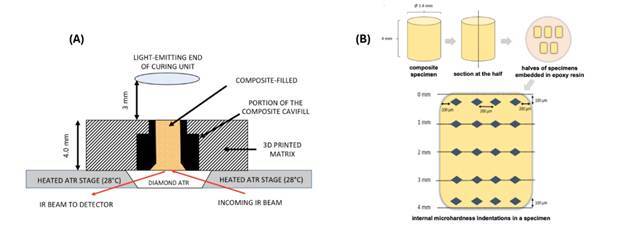
(A). Depiction of the specimen mold used to photopolymerize 4-mm thick bulk-filled increments of composite while obtaining real-time infrared spectra of the bottom composite surface. (B) Schematic representations of the sample preparation for microhardness measurements.

A bulk-filled composite (Tetric PowerFill, lot # Z024WR, shade IVA, Ivoclar Vivadent, Schaan, Liechtenstein) was tested for monomer conversion at 4 mm depth when irradiated with the curing tips of each type of light curing unit held approximately 3 mm from the top of the composite specimen. A special specimen mold was developed to help overcome the problem of the composite separating from the diamond attenuated total reflectance (ATR) element of the spectrometer during light exposure using the laser diode unit. Figure 1 (A) is a diagram that outlines important aspects of that mold.

In this method, the composite dispensing stopper end of a cavifil compule was removed, and all composite contents were discarded. A 15 x 12 x 4 mm tall block of plastic was 3D printed, containing the internal shape of the outline of the component in its middle. The empty cavity was inserted into the 3D printed mold so that the large end (where the plunger was located) was flush with the mold surface and held securely using a cyanoacrylate cement. The excess compule tube above the upper 3D printed matrix surface was trimmed to be flush with the top of the block, resulting in a smooth-walled specimen holder 4 mm high with an inner bore diameter of 4 mm and a lower bore of 5 mm (against the crystal).

The completed mold was placed on top of a horizontal ATR attachment (Golden Gate, high temperature ATR, SPECAC, Orpington, UK), and the stage was heated to 30°C using the dedicated computer-controlled stage heater. This temperature was selected because it is similar to that observed in vivo when the composite is placed against freshly light-cured dentin bonding ^21^.

The ATR attachment was positioned in the optical bench of a Fourier Transform infrared spectrometer (FTIR) (Invenio-R, Bruker Scientific LLC, Billerica, MA, USA). Prior to placement, a very thin layer of petroleum lubricant was applied to the inner walls of the cavifil tube to help reduce bonding of the composite to the walls of the container, thus eliminating lifting of polymerizing material from the diamond crystal surface during high-intensity light curing, resulting from polymerization shrinkage during light-curing.

The block mold was placed in a 3D printed jig that provided consistent placement on top of the ATR stage, with the bottom opening positioned directly in the center of the bottom surface mold aperture (Figure 1A). Unpolymerized composite (Tetric PowerFill, Ivoclar Vivadent) was added incrementally and compressed using a disc-shaped hand instrument of similar inner wall diameter until the composite was flush with the tip surface to be irradiated.

Spectral data were acquired using the chromatography mode of the FTIR, where two scans were averaged within 0.8 seconds at a resolution of 4 cm^-1^. At least 10 seconds of scans were acquired prior to light activation to provide an average value for the ratio of aliphatic-to aromatic C=C (1636 cm^-1^-to-1068 cm^-1^ peak height values, respectively) of each uncured specimen. Each type of light curing unit was held at a 3 mm distance from the tip of the composite surface and activated for the appropriate time interval using pre-set modes. The beginning of light output was detected using a silicon photodiode connected to a trigger connector box, the output of which was fed to the same time base as the infrared spectra were collected. Data were continuously collected for a period of 5 minutes after light exposure. Data were processed using software that exported the time-based ratio of aliphatic-to-aromatic C=C for each time segment as well as the status of the curing light output. The exported data were imported into templates made using a spreadsheet program (Microsoft EXCEL version 2110, Microsoft 365 for Enterprise, Microsoft Corporation, Redmond, WA, USA).

In that spreadsheet, the degree of monomer conversion was calculated using reference to a calibration curve developed using known molar ratios of methacrylate-based aliphatic and aromatic C=C molar monomer content and their respective absorption ratios. Graphical depictions of the time-based conversions were made with incorporated time tags of light activation. The degrees of monomer conversion at 5 minutes past initiation of light exposure were determined. Five replications for each test condition were made, and all specimens were tested in a randomized order concerning LCU (laser LCU or multi-peak LED LCU), and exposure duration (for the laser LCU: 1, 2, or 3 applications of the 1-s long exposure duration; and for the multi-peak LED LCU: 3 seconds (“3s mode”) and 20 seconds in “High mode”).

### Microhardness Test

Following monomer conversion analysis, the cured specimens were easily recovered from the custom jig and prepared for microhardness measurements that were taken internally, after sectioning. Composite specimens were longitudinally sectioned in half (Figure 1B), and only one of their halves was used for microhardness testing (n = 5). The halves of the sectioned specimens were embedded in epoxy resin cylinders (EpoxiCure, Buehler, Lake Bluff, IL, USA), and the composite sectioned surfaces were polished with SiC sandpaper (600, 1200, 2000-grit, 3M of Brazil, Sumaré, SP, Brazil) ^4^. The longitudinal and internal surface microhardness analysis was performed in five depths: 100 µm, 1 mm, 2 mm, 3 mm, and 390 µm. The microhardness indentations were performed using a microhardness tester (Future-Tech FM Corp, Tokyo, Japan) that was coupled to software (FM-ARS 9000, Future-Tech FM Corp), applying a static load of 50 g (0.49 N) for five seconds to each sample.

For the depths “100 µm” (the “top-most surface” was considered the top microhardness value) and “390 µm” (the “bottom-most surface” was considered the bottom microhardness value). Four indentations were made on the sectioned and polished surfaces for each depth of the samples: two indentations in the center and two at the peripheral sides of the half-samples. The centered indentation was spaced 200 µm from each other, and periphery measurements were located 100 µm from the outer surface (Figure 1B). The average of the four microhardness measurements was considered the microhardness value for each depth. An indication of how well the bottom surface polymerized relative to the top surface was provided in the Bottom-to-Top hardness ratios.

### Statistical Analysis

Statistical analysis for monomer conversion analysis consisted of a one-way ANOVA testing the effect of each type of light and its exposure duration (laser LCU for 1, 2, and 3 seconds, and multi-peak LCU for 3 and 20 seconds) on monomer conversion at 5 minutes after cessation of light exposure. Pair-wise mean post-hoc analysis was performed using the Tukey Test. The microhardness results were analyzed using a two-factor ANOVA (main factors of curing light/mode and composite depth). Tukey’s tests were used for pair-wise means comparisons. All statistical testing was performed at a pre-set alpha of 0.05. Statistical analysis was performed on a personal computer using software: SigmaPlot 11 (SigmaStat, Palo Alto, CA, USA).

## Results

### Diode Laser Curing Light Characterization

**Figure 2:**
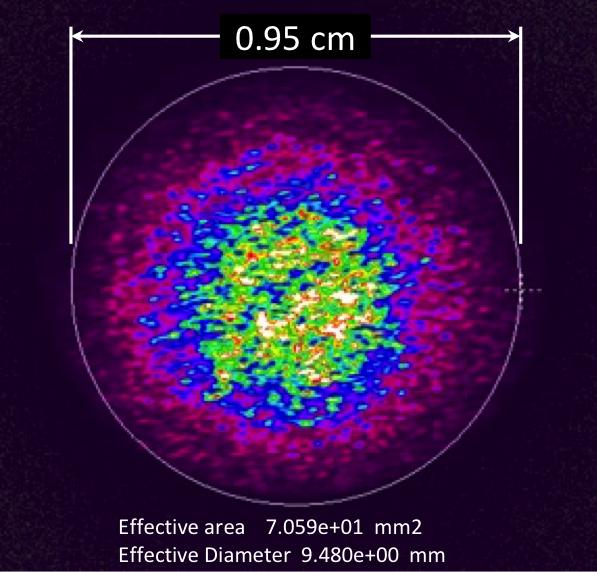
Dimensionally calibrated distribution of power within the Monet beam, highlighting the effective optical diameter of this curing light.

The effective area of the collimated beam from the diode laser LCU (Monet laser curing light) is displayed in Figure 2, obtained from the laser beam profile analysis software. From this figure, it can be seen that the power distribution within the beam is not uniform, with much higher irradiance values in the central core than toward the periphery. The edge of the symmetrical circumferential beam was easily identified using the imaging apparatus and indicated that the beam diameter measured 0.95 cm (0.71 cm^2^). These measurements were used for the determination of irradiance values of the Monet from spectral power values that were captured from radiometric data.

**Figure 3:**
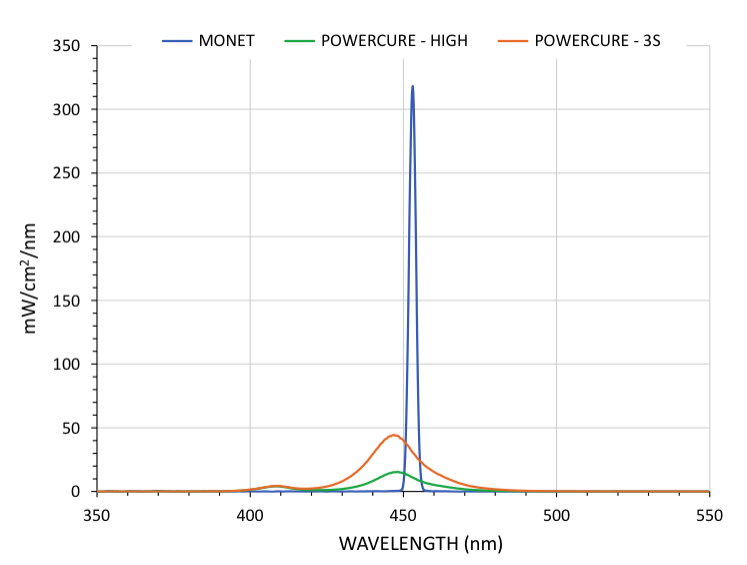
Spectral irradiance profile of the Monet laser diode curing light (blue trace) and PowerCure LCU used in its “High” output mode (green trace) and when used in its most powerful, “3s cure” mode (orange trace).

**Figure 4:**
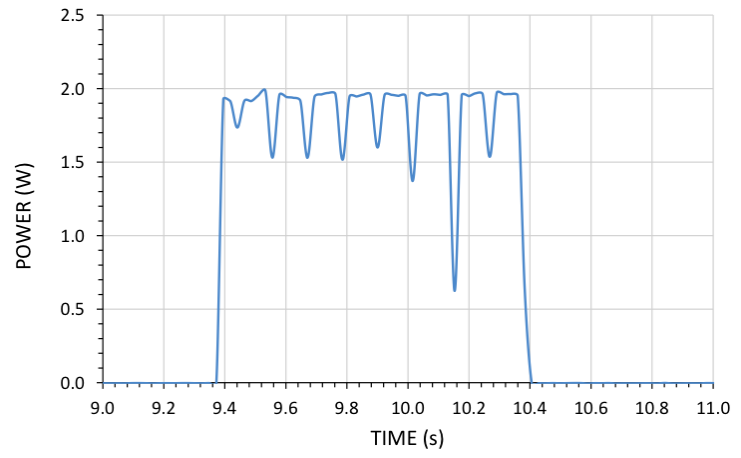
Example of captured power emission integrated between 430 to 480 nm using the STS-RAD spectroradiometer over the duration of a 1-second-long exposure.

The spectral irradiance of the diode laser curing light is displayed in Figure 3, in which the graph displays a very intense, narrow-banded light output symmetrically centered near 453 nm. The irradiance mean was 2,495 mW/cm^2^ (±17) between 350 and 550 nm (using 0.95 cm and the effective beam diameter). This laser curing unit generated the following values of radiant exposure for one, two, and three second curing times: 2.5 J/cm^2^, 5.0 J/cm^2^, and 7.5 J/cm^2^, respectively. When the high-speed data were displayed, it was evident that the output from the laser curing light is not continuous, but instead is pulsed. Also of interest was the fact that the peak power output is slightly less than 2 Watts (Figure 4).

### Multi-Peak LED Curing Light Characterization

In contrast, the spectral irradiance from the multi-peak LED-based light (Bluephase PowerCure) is much lower than the laser and provides two separate broad emission peaks. The spectral irradiance profiles of this light used in its “High” power mode and “3s cure” mode are seen overlaid in Figure 3. It can be seen that only the blue output at 449 nm is increased by changing the power output of this light-curing unit. The violet output at 408 nm remains the same for both output modes.

The average radiant emittance of the multi-peak LED LCU in its “High” output mode was 1,105 mW/cm^2^ (±1). Used for a 20-second-long exposure, this light provided a radiant exposure value of 22.3 J/cm^2^. In the “3s cure” mode, this LCU provided 3,099 mW/cm^2^, which correlated to a radiant exposure value of 9.3 J/cm^2^ over its exposure duration.

### Monomer Conversion Analysis

**Figure 5:**
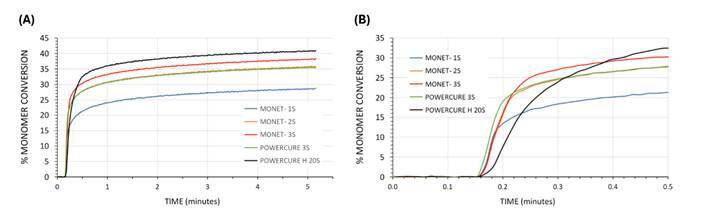
(A) Overlay of real-time monomer conversion at 4 mm composite depth captured throughout the light exposure and for a period of 5 minutes afterwards. (B) Scale enlargement of real-time conversion plot among lights, focusing on what was occurring very early on during each exposure type.

Figure 5 (A) provides examples of the real-time composite conversion values for all lights and curing times plotted on the same time axis for all light units and photopolymerization times and modes. An immediate and very steep rise during the initial light activation is noted for all groups. Following cessation of light exposure, conversion values continued to increase and slowly transitioned to appear as a more low-sloped, linear relationship with time. The highest conversion was seen throughout the scans when using the multi-peak LED LCU on “High” mode for 20s (black trace), followed by the diode laser curing light when applying three sequential 1-second-long exposures (green trace). The time/conversion traces for the multi-peak LED LCU used in its “3s cure” mode and the laser curing light used for two successive 1-second-long exposures seem to mirror themselves almost perfectly with time (green and orange traces). The lowest composite conversion values were observed when using the laser curing light for its default 1-second-long exposure (blue trace).

An enlarged image of composite curing at the very start of light exposure is seen in Figure 5 (B). Interestingly, all the conversion profiles using the laser curing light indicated similar slopes, with parallel traces breaking away after cessation of the light for 1 second (blue trace), for 2 seconds (orange trace), and for 3 seconds (red trace). The initial slope of the time-based conversion plot for the multi-peak LED LCU “3s cure” mode (green trace) occurred slightly before that of the laser light, and was parallel to that light as well. However, the start of conversion of the multi-peak LED LCU in its 20s “High” mode seemed delayed, and occurred at a much slower rate until 0.22 s (black trace).

Preliminary conversion data analysis indicated that both the normality and equal variance tests were passed, allowing further data analysis for the one-factor ANOVA to be performed. The results of that testing indicated that statistically significant differences were found among conversion values at 5 minutes after cessation of light-curing. The Tukey test was performed among group mean values to identify light/exposure groupings whose monomer conversion values were not significantly different.

**Figure 6:**
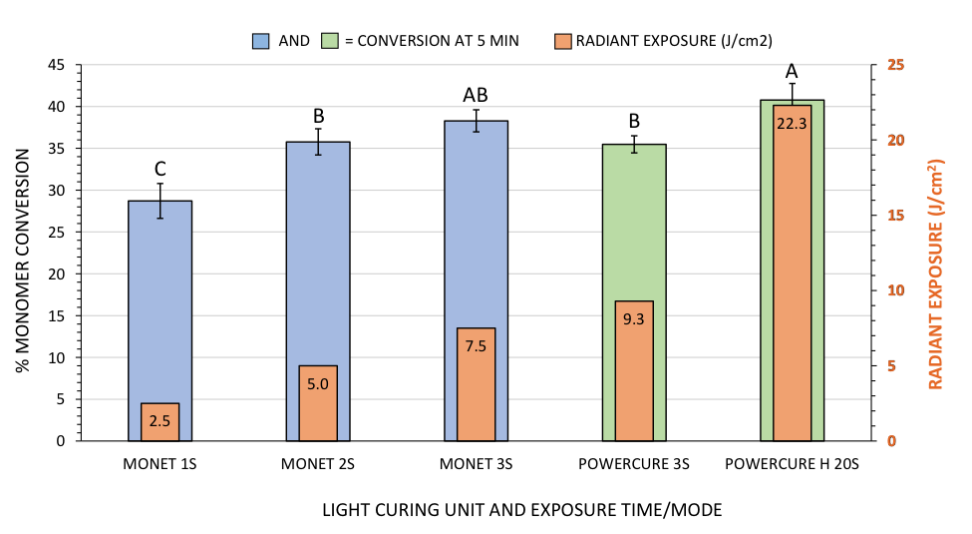
Graphical display of mean monomer conversion at 5 minutes from the end of light exposure among the various photocuring methods used. Vertical bar = ± 1 standard deviation. Monomer conversion values of bars identified using similar upper-case letters are not significantly different. Orange bars within blue or green colored bars indicate the labeled value of radiant exposure supplied to the specimen during photocuring (y-axis scale).

Figure 6 displays the mean monomer conversion value obtained at 5 minutes from the end of light curing, as well as an overlay graph of the radiant exposure that was applied to each specimen during the particular photocuring scenario. The data indicate a significant increase in composite monomer conversion at 4 mm depth when the duration of exposure from the diode laser curing light is increased from 1 to 3 seconds, an increase correlated with increasing radiant exposure as well. Comparing composite monomer conversion values when using the multi-peak LED LCU, the lower irradiance mode, but for a much longer exposure duration (“High” mode for 20 seconds at 1,105 mW/cm^2^), provided significantly greater conversion values than when the higher irradiance and shorter duration mode was applied (3 seconds at 3,3099 mW/cm^2^).

The diode laser curing light used for three sequential, 1-second-long exposures (a total of 7.5 J/cm^2^) yielded a composite degree of conversion value (around 38%) that did not statistically differ from multi-peak LED LCU (around 41%) when used in its “High” output mode for 20 seconds (22.3 J/cm^2^ of radiant exposure). Composite monomer conversion values at 4 mm deep with statistically similar results were found for the laser curing light, which accomplished this output using only 2 or 3 seconds of exposure (5.0 J/cm^2^ and 7.5 J/cm^2^), and the multi-peak LED LCU using “3s cure” mode that applied 9.3 J/cm^2^ to the composite (Figure 6).

### Microhardness Tests

**Figure 7:**
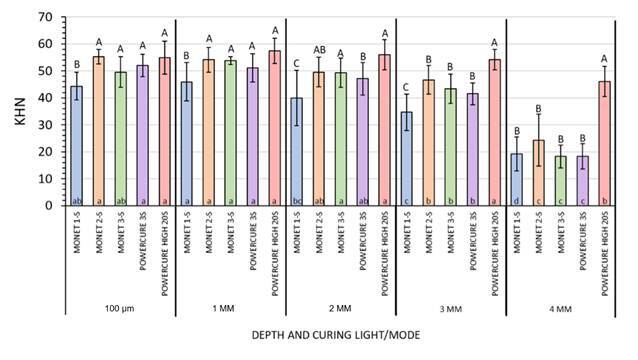
Mean microhardness (KHN) at composite depth according to light unit exposure duration, and output mode. Within a depth grouping, bars identified with similar “upper-case letters” (among lights, exposure durations, exposure modes) are not significantly different.

The data passed both normality testing (Shapiro-Wilk) and the equal variance tests, allowing parametric analysis to be performed. The two-factor ANOVA indicated that the major factors of “curing light/mode” and “composite depth” factors, as well as their interaction term, significantly contributed to microhardness values (p < 0.001). The mean microhardness values at each depth for each curing light condition and curing time are presented in Figure 7.

At the zero and 1-mm depths, the statistical grouping of test parameters was similar, with the 1-second long exposure to the diode laser curing light demonstrating a value significantly different from all others, which were not significantly different among themselves. At 2 mm depth, this trend shifted slightly, with distinctions being made among the longer laser curing light exposures and the multi-peak LED LCU “3s cure” mode. However, the 1-second-long laser curing light exposure remained significantly lower than all other test groups.

**Figure 8:**
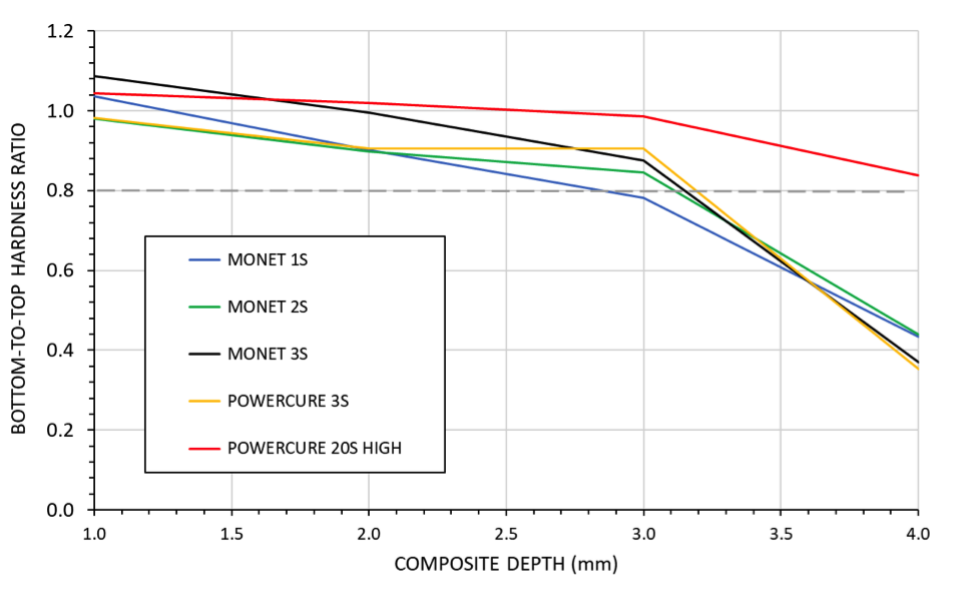
Average Bottom-to-Top microhardness values for each curing light/mode with respect to depth below the top surface. The grey horizontal dashed line at 0.8 indicates the bottom-to-top hardness ratio considered acceptable.

At 3 mm depth, the highest microhardness value was seen using the 20-s long multi-peak LED LCU “High” exposure, while the 1-s laser light exposure continued to provide the lowest microhardness. Hardness values of all remaining groups were not significantly different and fell between the high and low extremes. At 390 µm depth, the highest hardness value by far was seen using the multi-peak LED LCU in the “High” mode for 20s, while all the other curing conditions produced significantly lower hardness values and were not significantly different from each other (Figure 8).

Microhardness tended to decrease with increasing depth, regardless of the curing unit and time. Figure 8 presents these values, obtained by dividing the average hardness readings at each similar depth below the top surface by the average hardness values obtained for a given curing light/mode at a specified depth (considered as “bottom”). The findings of this interpretation indicate that the only light curing unit providing what is considered to be an acceptable bottom-to-top ratio at all depths up to and including 390 µm was the multi-peak LED LCU used in the “High” mode applied for 20s exposure (red trace). With the exception of the laser curing light used for 1 second (blue profile trace), all curing lights and modes provide a bottom-to-top ratio in excess of 0.8 at 3 mm depth. However, after that depth, all light-curing methods (except the multi-peak LED LCU in “High” mode used for 20 seconds) indicated bottom-to-top values that were greatly lower than the minimal acceptable value (Figure 8).

## Discussion

The first research hypothesis, that the composite monomer conversion at 4 mm depth varies according to the LCU types and curing modes used, was accepted. The light activation using short exposure times resulted in lower monomeric conversion than longer exposure times for both LCUs.

In this study, the average irradiance of the multi-peak LED LCU in its “High output mode” was 1,105 mW/cm^2,^ and when used for a 20-second long exposure, this light provided a radiant energy value of 22.3 J/cm^2^. Using the “3s cure” mode, this LED LCU provided 3,099 mW/cm^2^ of irradiance and a radiant exposure value of 9.3 J/cm^2^. These radiant exposure values provided by the multi-peak LED LCU were higher than those obtained for the diode laser curing light, even when it was used for a 3-second duration (7.5 J/cm^2^).

Tetric PowerFill resin composite contains photoinitiators based on camphorquinone (CQ), Lucirin TPO, and Ivocerin. CQ is a common photoinitiator with the optimal light absorption at 470 nm. Visible light absorption of the TPO photoinitiator ranges from 390 to 410 nm. Ivocerin presents a more absorption spectrum in the low wavelengths (390-445 nm) with a peak absorption at 408 nm. Ivocerin and TPO photoinitiators have been proven to be more reactive for high-power light-curing units than the standard CQ/amine photoinitiator system ^1^ ^,^ ^13^. The blue light from the diode laser curing unit is a narrow-banded light, near 453 nm, and has the capacity to activate CQ and Ivocerin initiators, and slightly the TPO one. On the other hand, the multi-peak LED-based light (Bluephase PowerCure) emits violet light at 408 nm-peak and blue light at 449 nm-peak, more compatible with three photoinitiators.

The degree of conversion of Tetric PowerFill bulk-fill composite obtained with laser LCU used for 3 seconds (around 38%) did not differ significantly from that of the multi-peak LED LCU used for 20 seconds in "High" mode (around 41%). However, the radiant exposure delivered from the diode laser LCU to the composite was 7.5 J/cm^2^ for 3 seconds, while the multi-peak LED LCU delivered 2.3 J/cm^2^ for 20 seconds. On the other hand, the radiant emittance mean of the diode laser LCU was 2,495 mW/cm^2^ for 3 seconds, with a peak output power of approximately 2 W, while the radiant emittance of the multi-peak LED LCU in its “High” output mode was 1,105 mW/cm^2^ for 20 seconds.

The tip diameter of the diode laser LCU is 11 mm, but the area of the light tip of the Monet laser, where the great majority of emission occurs, falls within only the very central portion of the lens. The spectral output is narrow-banded, and its effective output area of emission is 5.2 mm in diameter^17^. In this central area, the beam profile is very homogeneous in wavelength, very intense, and the diode laser LCU delivers 2,495 mW/cm^2^ of radiance emittance. Due to the collimation, narrow spectral output, and low output area of emission effects of the Monet curing light, multiple exposures to the restoration surface are necessary in extensive composite restorations (28-30).

To provide a perspective of the much higher irradiance the diode laser curing light has with respect to the multi-peak LED LCU, Figure 3 overlays the spectral irradiance profiles of all lights and curing modes graphed to the same Y-axis range. This figure places the relative radiant spectral irradiance values of these dental light-curing units in perspective. The output from the laser LCU is seen to be very concentrated about a central wavelength of 453 nm, whereas the blue output from the multi-peak LED LCU is much less and has a much broader spectral distribution than does the laser. As mentioned before, the violet output at 408 nm for the multi-peak LED LCU does not change with mode selection, and remains at a very low value, and is broadly distributed compared to the laser LCU, which has no light output within this spectral range.

The laser curing light is pulsed, and the peak power output is less than 2 Watts. Manufacturer-supplied information for the specific Monet curing light used in this study indicated that the power output was 1,97 Watts. This value is quite consistent with the peak output levels obtained in this high data rate capture image. Also, a study showed that the power output of the Monet curing unit was also 1,971 Watts, the total irradiance was 4,645 mW/cm^2,^ and the radiant exposures were 4.6 J/cm^2^ for a one-second exposure time and 13.9 J/cm^2^ for 3 seconds ^17^.

The composite polymerization rate at 5 minutes promoted by diode laser curing light for a 1-second curing time was lower than other curing modes from the LCUs. The 2.5 J/cm^2^ of radiant exposure delivered by laser light for 1 second also yielded the lowest composite monomer conversion at 5 minutes and 390 µm depth when compared with other curing modes of LCUs. The microhardness results for laser LCU using 1 second of exposure duration regarding other curing modes showed the same behavior, except for the 390-µm depth, in which the composite microhardness measured after curing with the multi-peak LED for 20 seconds yielded higher microhardness than the laser light used for 1 second. Other studies also showed that diode laser curing light used for 1 second produced the worst hardness and cure results for composites tested ^17^ ^,^ ^18^ ^,^ ^22^.

The second research hypothesis, that the composite microhardness reduces with increasing depth, regardless of the LCU and curing mode used, was also accepted. The composite microhardness at 1 mm depth cured by diode laser light for one second was reduced when compared to the microhardness close to the surface. When the composite was cured with the multi-peak LED LCU for 3 seconds (“3s cure” mode) and with the laser light for 2 and 3 seconds, the depth of cure of the composite remained stable until 2 mm. For the multi-peak LED LCU, the composite cured for 20 seconds yielded stable microhardness until 3 mm depth, and it showed the highest microhardness at the bottom of the samples (390 µm depth) among the curing modes of both LCUs tested in this study.

A study reported that the light transmission through the Tetric PowerFill composite was higher than that of the conventional resin composite ^13^, which light-cured with the fast high-intensity curing protocol resulted in inferior results for some important material properties ^31^. Also, a significant decrease in the amount of light transmitted as the thickness increased for five bulk-fill composites tested with multi-peak LED curing light (Bluephase Style, Ivoclar) or single-peak (Elipar S10, 3M) was reported. On the other hand, the effect of the curing unit on the hardness was minimal. The 1-, 2-, and 4-mm-thick specimens of SDR (Dentsply Sirona), X-tra Fill (Voco), and Filtek Bulk Restorative (3M) composites achieved a hardness bottom/top ratio of approximately 80% when either curing unit was used ^31^. Another study showed that little light reached the bottom of proximal box regions when the light tip was positioned at the center of restorations. Also, it recommends that curing lights with wide tips, homogeneous light beam profiles, and longer exposure times are preferred when light-curing extensive MOD restorations ^28^.

A study assessed the influence of the same 3-second multi-peak LED LCU on the mechanical properties and degree of conversion of four bulk-fill composites (Tetric PowerFill/Ivoclar; Tetric PowerFlow/Ivoclar, Filtek One Bulk Fill Restorative/Solventum, and SDR Plus Bulk Fill Flowable/Sirona Dentsply). Rapid 3-second light curing reduced the flexural modulus and yielded a lower degree of conversion in deep areas (at the bottom of 4 mm-high samples) of all tested bulk-fill composites ^14^. Other studies showed that the effects of rapid 3-second multi-peak LED LCU depended on the type of resin composite tested, and the flowable resin-based materials seemed to be more affected than incremental and sculptable bulk-fill ones ^12^. Garoushi et al. ^13^ ^)^ evaluated the influence of 3- and 20-second light curing times on some physical properties of two hybrid composites (Tetric PowerFill/Ivoclar and Essentia U/GC Corp.). Results suggested that the shrinkage stress, degree of conversion, and hardness of both composites significantly increased with the conventional 20-second light curing time, while light curing conventional composite resin with a rapid 3-second light curing protocol resulted in inferior results for some important material properties.

The main advantage of high-power LCUs has been related to the reduction of the resin-based dental materials’ curing time. However, evidence showing that rapid 1 to 3 seconds and high-power units are adequate clinical options for light curing is still undetermined, as some studies have reported the possibility of a low depth of cure ^12^ ^,^ ^14^ ^,^ ^17^ ^,^ ^18^ ^,^ ^22^. The multi-peak LED LCU in its “High” mode delivered 1,105 mW/cm^2^ of irradiance output and, when used for 20 seconds, provided a radiant energy value of 22.3 J/cm^2^, which yielded the best results of monomer conversion and microhardness at the bottom of composite samples.

A low degree of monomeric conversion at 4 mm depth can lead to an early failure at the adhesive-composite interface, which can compromise the longevity of composite restorations ^4^ ^,^ ^17^ ^,^ ^25^ ^,^ ^26^ ^,^ ^27^ ^,^ ^28^ ^,^ ^31^. Also, there is concern about the toxicity of the uncured composite, and studies have investigated this clinical impact ^32^ ^,^ ^33^. To improve the degree of conversion and mechanical properties of composites, restorations must be performed by dentists trained to apply the restorative composite in deep cavities properly. Dentists must know how to select a good light-curing unit, a proper curing mode, a type of resin composite, a curing time, and how to use it intraorally, according to each clinical case ^28^ ^,^ ^29^ ^,^ ^30^ ^,^ ^31^. Also, studies have demonstrated that the importance of a high radiant exposure, with longer or standard exposure times, can be used to increase the depth conversion of the restorative composite, compared to shorter or high-irradiance protocols ^17^ ^,^ ^20^ ^,^ ^25^ ^,^ ^29^ ^,^ ^30^ ^,^ ^34^.

The dentists should be aware of the technical details of light-curing units, such as wavelengths of light, radiant exposure delivered, curing modes, and exposure times, as well as the characteristics, indications, and compositions of restorative composites. The multi-peak LED LCU presents a 3-second cure program that delivers more than 3,000 mW/cm^2^ and must be used with a specific commercial bulk-fill composite (Tetric PowerFill/Ivoclar). On the other hand, this resin composite does not present a specific indication for use with diode laser curing light.

## Conclusion

According to the results of this study, it was possible to conclude that:

1. Radiant exposure delivered to the composite is an important requirement to achieve a high depth of cure. PowerCure unit used for 20 seconds provided a bottom-to-top hardness ratio of 0.84, indicating sufficient curing at depth and a higher degree of conversion at 4 mm depth.
2. The top surface microhardness of the composite did not differ among curing modes, except for the laser light used for one second, which showed the lowest microhardness value.
3. The composite microhardness reduced with increasing depth, regardless of the curing unit and time used.
